# A Short History of Medicine, from the Earliest Periods up to the Revival of Literature

**Published:** 1850-08

**Authors:** 


*A Short History of Medicine*, from the earliest periods up to the revival of literature. An Introductory Lecture delivered March 28, 1850, in the Philadelphia College of Medicine, by James Bryan, M. D., Prof. of Institutes and Medical Jurisprudence.

We have neglected making acknowledgments for this interesting and instructive discourse, owing to its having been received during our absence, and mislaid. It is chiefly confined to the history of ancient medicine, a field of curious and useful research, too much neglected by physicians, albeit, our profession is so often charged with a tenacious adherence to antiquity. Prof. Bryan, in addition to his connection with Geneva College, has lately been appointed to a chair in the Philadelphia College of Medicine.

